# Supplementary material for: A protocol for chemical competence in phytopathogenic Ralstonia
Source: Access Microbiol. 2026 Mar 23;8(3):001135.v3. doi: 10.1099/acmi.0.001135.v3 (PMC13008376; doi:10.1099/acmi.0.001135.v3)
Supplement: Supplementary Material 2. [file acmi-8-01135-s002.pdf]

Supplemental Figures for “A protocol for chemical competence in phytopathogenic *Ralstonia*”

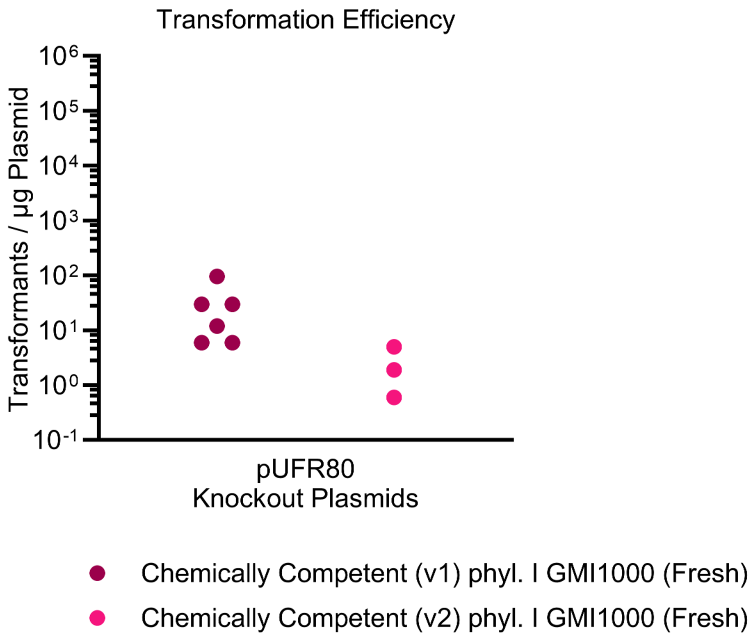

Figure S1. Transformation efficiencies for chemical competence in the *Ralstonia* strain GMI1000 using pUFR80 knockout plasmids. The symbols represent individual transformations with symbol color representing the protocol variant.

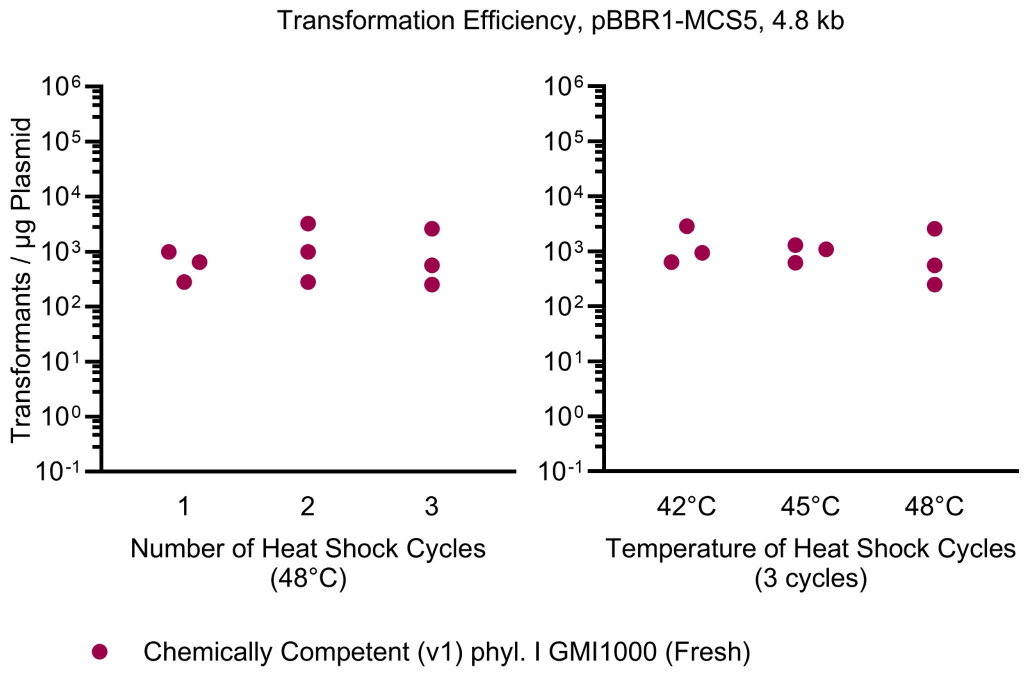

Figure S2. Transformation efficiencies for chemical competence in the *Ralstonia* strain GMI1000 using the plasmid pBBR1-MCS5 with varying heat shock cycles and temperatures. The symbols represent individual transformations. All transformations shown here were performed using the preliminary protocol.
